# Supplementary material for: Splice-Junction-Based Mapping of Alternative Isoforms in the Human Proteome
Source: Cell Rep. Author manuscript; Available in PMC 2020 Jan 15. (PMC6961840; doi:10.1016/j.celrep.2019.11.026)
Supplement: 2 [file NIHMS1546469-supplement-2.zip › DF1/PXD010154/Esophagus/Esophagus_31_SRSF11_VTAQPDVLEVQAEYITAGPGPSGGPGGGG.pdf]

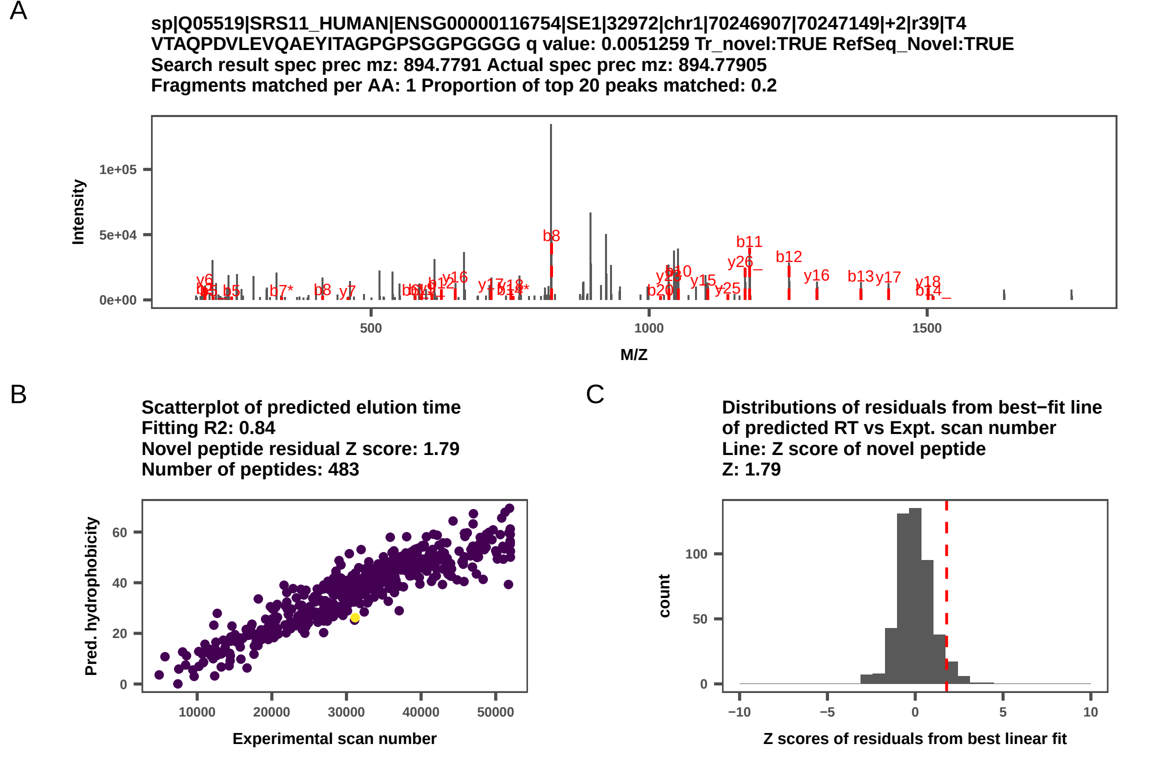

A
sp|Q05519|SRS11_HUMAN|ENSG00000116754|SE1|32972|chr1|70246907|70247149|+2|r39|T4
VTAQPDVLEVQAEYITAGPGPSGGPGGGG q value: 0.0051259 Tr_novel:TRUE RefSeq_Novel:TRUE
Search result spec prec mz: 894.7791 Actual spec prec mz: 894.77905
Fragments matched per AA: 1 Proportion of top 20 peaks matched: 0.2
Intensity
1e+05
5e+04
0e+00
b8
b11
b12
y26
y16
b13
y17
y18
b14
b20
y15
y25
b7*
y7
y6
b5
b12
y16
500
1000
1500
M/Z
B
Scatterplot of predicted elution time
Fitting R2: 0.84
Novel peptide residual Z score: 1.79
Number of peptides: 483
Pred. hydrophobicity
60
40
20
0
10000
20000
30000
40000
50000
Experimental scan number
C
Distributions of residuals from best−fit line
of predicted RT vs Expt. scan number
Line: Z score of novel peptide
Z: 1.79
count
100
50
0
−10
−5
0
5
10
Z scores of residuals from best linear fit
